# The Role of Intergenerational Relationships: Applying the Family Stress Model to Grandfamilies

**DOI:** 10.1093/geroni/igaa057.1124

**Published:** 2020-12-16

**Authors:** Rachel Scott, Danielle Nadorff, Loriena Yancura, Melissa Barnett

**Affiliations:** 1 Mississippi State University, Starkville, Mississippi, United States; 2 Mississippi State University, Mississippi State, Mississippi, United States; 3 University of Hawai’i, Honolulu, Hawaii, United States; 4 The University of Arizona, Tucson, Arizona, United States

## Abstract

The Family Stress Model (FSM) of Economic Hardship (Conger, Rueter, & Conger, 2000) was developed to explain the impact of financial stress on families through links between economic difficulties, parental emotional distress, marital conflict, disrupted parenting behaviors, and child maladjustment. The FSM has been cross validated in samples of custodial grandparents (i.e., grandparents who provide substantial care for their grandchildren; Smith et al., 2017). The current study modified the FSM by replacing inter-parent relationship difficulties with inter-generational relationship problems between the custodial grandparents and their children to ultimately examine the adjustment of the grandchildren. This change to the model is supported by prior research conducted on intergenerational stress impacting the parenting and subsequent development of children in grandfamilies (Barnett, Mills-Koonce, Gustafsson, & Cox, 2012). Using a nationwide sample of 317 custodial grandparents aged 40 and older (M = 61 yr) the fit of the modified model was tested using AMOS 26. Latent variables in the model included Economic Pressure, Caregiver Distress, Disrupted Parenting, Intergenerational Relationship, and Child Adjustment. Moderate fit was achieved (χ2(308) = 574.88; CFI = .896; RMSEA = .052). All pathways were significant with the exception of Disrupted Parenting to Child Adjustment. These results indicate that intergenerational relationships are an important predictor of child adjustment, and an applicable substitute for inter-partner relationships when modeling family stress in custodial grandfamilies. Details and clinical implications will be discussed.

